# *Plasmodium falciparum* antigenic variation. Mapping mosaic *var* gene sequences onto a network of shared, highly polymorphic sequence blocks

**DOI:** 10.1111/j.1365-2958.2008.06248.x

**Published:** 2008-06

**Authors:** Peter C Bull, Caroline O Buckee, Sue Kyes, Moses M Kortok, Vandana Thathy, Bernard Guyah, José A Stoute, Chris I Newbold, Kevin Marsh

**Affiliations:** 1 Kenya Medical Research Institute Centre for Geographic Medicine ResearchCoast, Kilifi, Kenya; 2 Nuffield Department of Clinical Medicine, University of OxfordJohn Radcliffe Hospital, Oxford, UK; 3 Department of Zoology, University of OxfordOxford, UK; 4 US Army Medical Research Unit-KenyaKisumu, Kenya

## Abstract

*Plasmodium falciparum* erythrocyte membrane protein 1 (PfEMP1) is a potentially important family of immune targets, encoded by an extremely diverse gene family called *var*. Understanding of the genetic organization of *var* genes is hampered by sequence mosaicism that results from a long history of non-homologous recombination. Here we have used software designed to analyse social networks to visualize the relationships between large collections of short *var* sequences tags sampled from clinical parasite isolates. In this approach, two sequences are connected if they share one or more highly polymorphic sequence blocks. The results show that the majority of analysed sequences including several *var*-like sequences from the chimpanzee parasite *Plasmodium reichenowi* can be either directly or indirectly linked together in a single unbroken network. However, the network is highly structured and contains putative subgroups of recombining sequences. The major subgroup contains the previously described group A *var* genes, previously proposed to be genetically distinct. Another subgroup contains sequences found to be associated with rosetting, a parasite virulence phenotype. The mosaic structure of the sequences and their division into subgroups may reflect the conflicting problems of maximizing antigenic diversity and minimizing epitope sharing between variants while maintaining their host cell binding functions.

## Introduction

Children living in malaria endemic areas develop significant naturally acquired immunity to severe malaria during the first 5 years of life (Marsh, 1992). The variant surface antigens (VSA) expressed on malaria-infected erythrocytes are strong candidate targets of naturally acquired immunity as they are exposed to host antibodies for long periods while the parasite is still alive. The major component of VSA, called PfEMP1 (*P. falciparum* erythrocyte membrane protein 1) is encoded by a family of approximately 60 *var* genes per genome (Baruch *et al*., 1995; Smith *et al*., 1995; Su *et al*., 1995). These molecules are implicated as virulence factors. Through interactions with host molecules such as ICAM-1, CD36, CR1 and CD31, PfEMP1 plays a central role in mediating cytoadherence of infected erythrocytes to host cells. Cytoadherence is believed to be responsible for the severe pathology associated with *P. falciparum* malaria (Craig and Scherf, 2001; Kyes *et al*., 2001; Baruch *et al*., 2002; Flick and Chen, 2004). PfEMP1 molecules undergo clonal antigenic variation meaning that a single genotype can evade host antibodies by switching between *var* genes (Roberts *et al*., 1992). After repeated exposure to infection, a repertoire of antibodies build up that can recognize most VSA circulating in the parasite population. The gradual restriction of the PfEMP1 molecules capable of sustaining infection as the host antibody repertoire develops could potentially explain the observed modification of the host parasite relationship that occurs during the development of naturally acquired immunity to malaria (Bull *et al*., 1998; Giha *et al*., 2000).

*var* genes have a modular organization (Smith *et al*., 2000; Gardner *et al*., 2002; Lavstsen *et al*., 2003) consisting of various numbers and combinations of duffy binding-like (DBL) domains of different types (α, β, δ, ε, γ and x) and cysteine rich interdomain regions (CIDR), again of different classes (α, β, γ). The overall architecture of the genes is highly variable both in terms of the total number of domains and their order within the molecule.

This architectural diversity is generated at least in part through recombination between *var* genes on non-homologous chromosomes (Freitas-Junior *et al*., 2000; Taylor *et al*., 2000a). As a result *var* genes at homologous positions within the genome of two different isolates can have very different architectures (i.e. combinations of DBL and CIDR domains; Kraemer *et al*., 2007). Recombination or gene conversion have also led to the generation of considerable mosaicism within *var* gene domains (Ward *et al*., 1999; Taylor *et al*., 2000a). Recombination and gene conversion are commonly used approaches for generating genetic diversity in the surface proteins of protozoan and bacterial pathogens (Deitsch *et al*., 1997; Santoyo and Romero, 2005), many of which have a mosaic structure. (Gibbs *et al*., 1989; McGraw *et al*., 1999; Urwin *et al*., 2002; Brayton *et al*., 2002; Haake *et al*., 2004; Mauricio *et al*., 2007). Because the constituent sequence segments of such genes may have different evolutionary histories, standard phylogenetic analysis that assumes simple, tree-like relationships between the genes may be inappropriate (Holmes *et al*., 1999).

Very little is still known about how *var* genes are organized in natural parasite populations. It is possible that specific *var* gene sequences ‘types’ exist that are associated with particular combinations of DBL and CIDR domains which, owing to their combined cytoadherence characteristics, are more likely to lead to the development of severe malaria. However, if non-homologous recombination is common between all *var* genes, then sequence markers defining such genes may be impossible to find. A third possibility is that some non-homologous recombination events are more favoured than others. This could occur, e.g. if the genomic organization of the gene family, or the architecture of individual genes made some genes more likely to form chiasma during meiosis. If this was the case, then identification of groups of genes that tend not to recombine with one another might help in the identification of pathologically important subsets.

This third scenario appears to be the most accurate. The complete sequencing of a single *P. falciparum* genome 3D7, uncovered the genomic organization of *var* genes in a single genome. The organization of *var* genes in two other laboratory isolates is also close to completely described (Kraemer *et al*., 2007). The genomic organization of *var* genes appears to reflect both their functional and immunological properties (Gardner *et al*., 2002; Robinson *et al*., 2003). A subset of *var* genes called ‘group A’, associated with a specific upstream sequence ‘upsA’, are transcribed in the opposite direction to the majority of *var* genes and appear to lack the normal capacity to bind to the host molecule CD36. (Kraemer and Smith, 2003; Robinson *et al*., 2003; Kraemer *et al*., 2007). This same group of genes also appears to have distinct immunological properties, being better recognized by naturally acquired antibodies carried by children growing up in a malaria endemic area (Jensen *et al*., 2004).

Because of the diversity of *var* genes there are limited positions within the molecules that can be reliably amplified and sequenced from clinical parasite isolates. As a result, several studies of clinical isolates have relied on samples of short ∼350 nt ‘sequence tags’ rather than on whole gene sequences. These sequence tags are amplified from priming sites within DBLα domains, one of the few domains that is present in most *var* genes (Taylor *et al*., 2000b). Many hundreds of these DBLα sequence tags are now available from parasites sampled worldwide (Taylor *et al*., 2000b; Kirchgatter and del Portillo, 2002; Bull *et al*., 2005; Albrecht *et al*., 2006; Kyriacou *et al*., 2006; Barry *et al*., 2007; Normark *et al*., 2007).

A simple way of classifying DBLα sequence tags is by dividing them into those containing two cysteine residues (cys2) and those (the majority) containing four cysteine residues (cys4) and a small minority containing 0, 1, 3, 5 or 6 cysteines (cysX). Based on genome sequence data most (but not all) cys2 sequences are from a putative subtype of DBLα domain called ‘DBLα1’ found in group A *var* genes, whereas all cys4 sequences are from non-group A *var* genes (Robinson *et al*., 2003). This distinction appears to be useful and two studies have reported associations between expression of cys2 *var* genes and severe malaria (Kirchgatter and del Portillo, 2002; Kyriacou *et al*., 2006). These data have been used to support the idea that group A *var* genes are associated with severe malaria. However, an exclusive role for group A *var* genes in severe malaria is challenged by studies using group-specific real-time PCR (Kaestli *et al*., 2006; Rottmann *et al*., 2006).

To understand more about the relationships between *var* genes from clinical isolates, we sought a simple non-phylogenetic approach to visualize the sharing of polymorphic blocks of sequence between large collections of DBLα tags. Various approaches have been used previously to detect, describe or account for recombination in small samples of *var* gene sequence (DePristo *et al*., 2006) or in the relatively conserved *var*2CSA gene implicated in malaria in pregnancy (Trimnell *et al*., 2006; Bockhorst *et al*., 2007). A recent study has used the Alignment Comparison Tool (Carver *et al*., 2005) to identify segments of whole *var* genes that are shared between different genomes 3D7, HB3 and IT4 (Kraemer *et al*., 2007). These methods tend to work well when there is a relatively low density of recombination breakpoints or with small collections of sequences, but are not suitable for the analysis of large collections of genes such as *var* where there has been a long history of non-homologous recombination and diversification.

The method we show here uses software designed for the analysis of large social networks to account for the fact that *var* genes interact with one another over time leading to the sharing of blocks of polymorphic sequence. Here, we have used this approach to address the following specific questions: (i) What are the major groups of *var* genes that share sequence blocks with each other? (ii) How do these relate to previously defined groupings? (iii) To what extent are these groupings maintained in *var* sequences collected worldwide? (iv) To what extent do groupings help us define functional groups of genes?

## Results

### Rationale and optimization of the network analysis approach

**Fig. 1:**
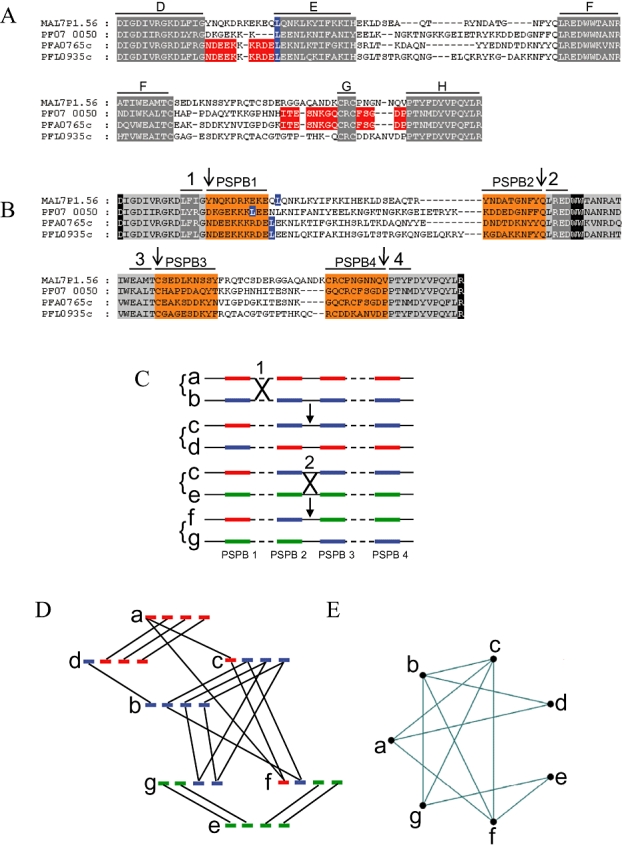
The rationale for the approach. A and B. A Clustal alignment of four *var* sequences from 3D7 genome. Comparison of genes would normally be based on an alignment of the regions that are most shared between different sequences. The alternative used here (B) is to align polymorphic blocks of sequence (orange) to fixed reference points that are known to be invariant (black shaded sequence). For this, alignment of some conserved residues (one example is highlighted in blue) takes lower priority than alignment with respect to the chosen anchor points. These ‘position specific polymorphic blocks’ (PSPBs) are defined at up to four positions, PSPBs1–4. The default start positions (positions closest to the anchor point) are shown with vertical arrows. We chose default positions for the PSPBs that were adjacent to, but did not overlap with previously defined ‘positions of limited variability’ (PoLV1-4) marked 1, 2, 3 and 4 respectively (Bull *et al*., 2005; 2007). C–E. A hypothetical recombination network. Two hypothetical recombination events (1 and 2) are shown (C) together with a summary of the PSPBs that would be shared between the recombining genes and their products (D), and the resulting recombination network that would be obtained if all the products of recombination were sequenced (E). Each line a-g represents a portion of a chromosome corresponding to the sequenced regions of a several hypothetical *var* genes. Black portions of each line represent the three islands of homology D, F and H (A) that were used as anchor points. Thick coloured portions represent the four position specific polymorphic blocks (PSPBs) used in the analysis as distinct markers for ancestral *var* gene fragments. Dotted portions represent regions that were not used to align sequence. The first recombination event (1) between variants a and b occurs between PSPB1 and PSPB2 giving rise to two different *var* gene variants c and d. Recombination of one of these products c with another variant e between PSPB2 and PSPB3 (event 2) generates variants f and g. The relationships between each of the seven variants can be expressed as a network (E). Though variants a and b share no PSPBs they are connected indirectly through sharing PSPBs with the *var* sequences that resulted from recombination event 1. The same can be said for variants c and e in relation to recombination event 2. Thus all the hypothetical genes shown in (C–E) could be considered to be in the same community of genes that are capable of sharing blocks of sequences through recombination.

The rationale for our approach is shown in Fig. 1. Sequence mosaicism within *var* gene sequences can easily be recognized through visual inspection of sequences. Blocks of sequence are frequently shared between two otherwise dissimilar sequences within regions of the sequence that are normally highly polymorphic (see Fig. 1A for an example).

DBLα sequence tags are highly variable in length and alignment of sequences by standard approaches relies on the introduction of gaps (Fig. 1A). To overcome the problem of inaccurate sequence alignment and assignment of location within such sequences we restricted the analysis to ungapped polymorphic sequence blocks at locations within *var* sequence tags that were fixed relative to one of three conserved anchor points, one at each end and one in the middle (highlighted with a black background in Fig. 1B). As shown in Fig. 1B this provided four independent window positions. Using only sequence within these windows we then simply asked whether two sequences were identical within any one of the sequence blocks. In this way, instead of regarding each sequence as a single highly diverse unit (Barry *et al*., 2007; Fig. 1A), we analysed them as multiple independent blocks of sequence each acting as genetic markers for the sequence to which they are anchored (Fig. 1B). The approach is therefore an *ad hoc* one that does not rely on assumptions of any particular model of evolution. This is an advantage at this stage of the analysis when so little is known about how diversity in these molecules is generated.

Henceforth we will refer to each sequence block as a ‘position specific polymorphic block’ (PSPB) (Fig. 1B). We used PSPBs to construct networks in which sequences are represented by nodes (vertices) that are joined by lines (edges) if they are identical at one or more of their constituent PSPBs (Fig. 1B–E).

We tested the approach using 1420 sequences. The majority of these (1228) were collected from 21 children from Kilifi, Kenya. These were supplemented with a worldwide collection of DBLα tag sequences 102 group A *var* genes (Trimnell *et al*., 2006). We will refer to these as ‘group A reference sequences’. Sixty-one *var* gene sequences from the complete genome sequence of a single parasite isolate 3D7 were included together with 29 *var*-like sequences from the chimpanzee malaria parasite *P. reichenowi*. We will refer to this as the ‘Kilifi network’.

**Fig. 2:**
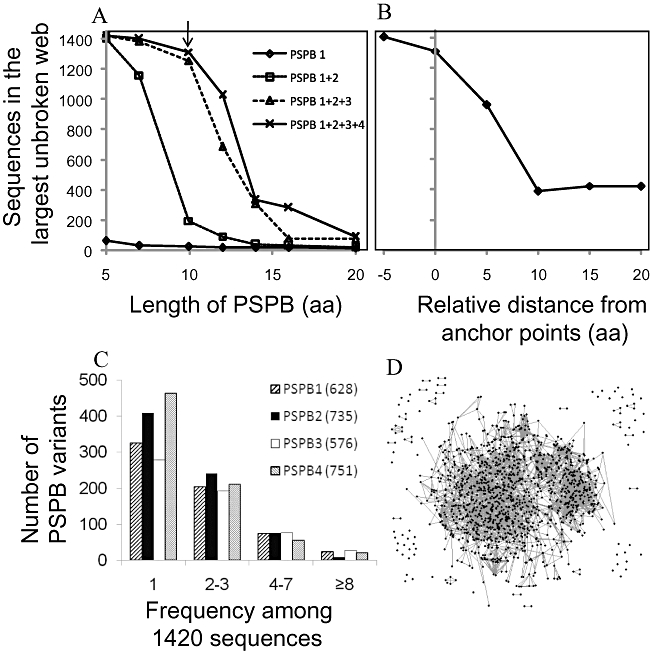
Optimization of the approach. A and B. Variation in network structure with length, number and position specific polymorphic blocks (PSPBs). (A) The largest number of sequences that form an unbroken network (giant component) was determined for different numbers of PSPBs (1–4) and different PSPB length. PSPB length was varied while the distance between the proximal ends of the PSPBs (their start positions) and their respective anchor residues were kept constant. Start positions are indicated with vertical arrows in Fig. 1B. (A) shows representative results for PSPB1 alone, PSPB1 + 2, PSPB1 + 2 + 3 and all four PSPBs. (B) The effect of varying the distance between the start positions and the anchor positions. Distances are shown relative to the default positions used in (A) and shown in Fig. 1B. For this analysis, we used four PSPBs and a window length of 10 aa. C. The frequency of each variant observed at PSPBs1–4 was determined among the 1420 sequences from the Kilifi network. A large proportion of PSPB variants only occurred once among these sequences. D. The basic structure of the Kilifi network containing 1420 sequences and constructed using four PSPBs and the default PSPB start positions shown in Fig. 1B. See Fig. S1 for the structures of networks drawn with only three PSPBs.

To determine the optimal conditions to perform the analysis we tested the extent to which the sequences tended to form an unbroken network (as in Fig. 1E) when the number of PSPBs used and their length and position were altered. For each set of conditions we counted the number of sequences that together formed the largest unbroken network of vertices and edges (henceforth called the ‘giant component’). Figure 2A shows that four PSPBs were sufficient to join the sequences together into a single giant component. The size of the giant component grows as more PSPBs are used because this provides more opportunities for a match between sequences. The size of the giant component diminishes as the PSPB length increases because longer sequences are less likely to remain intact over time. When using four PSPBs a PSPB length of 10 amino acids was about the longest that could be used before the size of the giant component began to reduce dramatically (indicated with an arrow on Fig. 2A). Using these parameters, we explored the effect of moving the positions of the PSPBs relative to the conserved anchor points. The size of the giant component diminished as the distance from the anchor points increased. This was expected because exact matches in the PSPBs of two sequences can be disrupted by both mutations within the PSPB itself and the introduction of insertions and deletions (indels) into the sequence between the PSPB and the anchor point. The greater the distance between the PSPB and the anchor point, the greater the opportunity for indels to disrupt a match. Based on these observations we chose to explore further the network derived from four 10 aa PSPBs. As shown in Fig. 2C 10 aa PSPBs were extremely diverse.

Under the chosen conditions 92% of the 1420 sequences were linked together within a single giant component. We used standard algorithms (see *Experimental procedures*) to cluster the vertices so that those that tended to share PSPBs with one another would be located in the same region of the network. The result is shown in Fig. 2D. Two major lobes were apparent, one small (on the right) and the other large. (See Supporting Folder S1 for three-dimensional views of networks shown).

### Comparison with previously defined groups of sequences

**Fig. 3:**
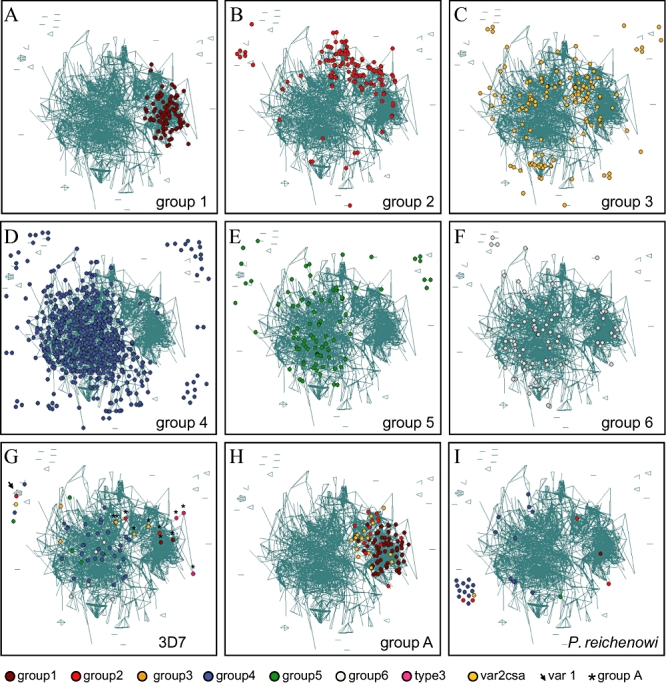
Locations of different groups of sequences within the Kilifi network. A–F. Locations of sequences falling in each of 6 previously defined cys/PoLV groups (Bull *et al*., 2005). G. Locations of 3D7 genes (Gardner *et al*., 2002). ‘Group A’ genes (genes associated with an upsA upstream element) are highlighted with an asterisk (*). *var1* is indicated with an arrow. However, *var1* is dimorphic within the tag region (Bull *et al*., 2005). The other *var1* sequence type, present in parasite line FCR3, is in cys/PoLV group 1 and shares PSPBs with other cys/PoLV group 1 sequences within the small lobe of the network (data not shown). H. Location of 102 group A reference sequences (Trimnell *et al*., 2006). I. Location of DBLα sequences from a *P. reichenowi* (chimpanzee malaria) genome (Wellcome Trust Sanger Institute).

Together with the number of cysteines present within the sequence tag, we previously used sequence features at specific positions called ‘positions of limited variability’ (PoLV) to classify DBLα sequence tags into six ‘cys/PoLV’ groups (Bull *et al*., 2005; 2007; see *Experimental procedures*). Cys/PoLV group 1, 2 and 3 sequences are cys2 sequences whereas cys/PoLV group 4 and 5 are cys4 sequences and cys/PoLV group 6 sequences are cysX sequences. In Fig. 3, colours are used to highlight the previous classification given to each of the sequences. Cys4 sequences are almost entirely absent from the small lobe of network (Fig. 3D–E). The vast majority of vertices within the small lobe represent cys2 sequences. Figure S2 summarizes how blocks of sequence are shared between cys/PoLV groups.

We next compared how *var* gene sequences obtained from a single isolate of *Plasmodium falciparum*, 3D7, map within the network (Fig. 3G). The following observations can be made. First, the tag region from the DBLα-like domain of the *var2csa* gene is found outside the giant component of the network. Expression of *var2csa* is found to be associated with placental malaria (Salanti *et al*., 2004). Second, the *var1* tag from 3D7 also lies outside giant component and is indicated with an arrow. This gene is highly conserved (Rowe *et al*., 2002; Winter *et al*., 2003). Third, the tag regions from the three Type 3 *vars* carried in the 3D7 genome lie at the periphery of the small lobe of the network. Type 3 *vars* are a short, highly conserved subset of group A genes. Neither *var2csa* nor Type 3 sequences can be amplified by the primers used in this study. Fourth, the group A sequences from 3D7 fall within or close to the small lobe of the network (marked with asterisks).

Figure 3H shows that this is a general characteristic of group A sequences. The global collection of 102 group A reference sequences are highly localized despite not falling exclusively within a single cys/PoLV group. Thus, cys/PoLV grouping and network mapping of sequences appear to complement one another as analytical approaches. The fact that group A reference sequences lie in a specific region of the network supports the idea that recombination between group A and non-group A genes is restricted (Kraemer and Smith, 2003; Kraemer *et al*., 2007).

Given the overall tendency of cys2 DBLα sequence tags to be associated with group A *var* genes (Robinson *et al*., 2003), it is interesting to contrast the tight localization of group A reference sequences with the much less tight localization of cys2 sequences, specifically cys2 sequences in cys/PoLV groups 2 and 3. This suggests that group A *vars* are a relatively genetically isolated subset of cys2 sequences.

Finally, Fig. 3I shows how DBLα*var* sequences from a single isolate of chimpanzee malaria parasite *P. reichenowi* lie within the network. Though only 14/29 (49%) of *P. reichenowi* sequences were retained within the giant component, the fact that any of these *P. reichenowi* sequences fell within the network was unexpected. Furthermore, *P. reichenowi* sequences are present within the network in positions consistent with their cys/PoLV group. This suggests a long-standing relationship between different subsets of *var* genes.

### An exploration of block-sharing groups within the network

**Fig. 4:**
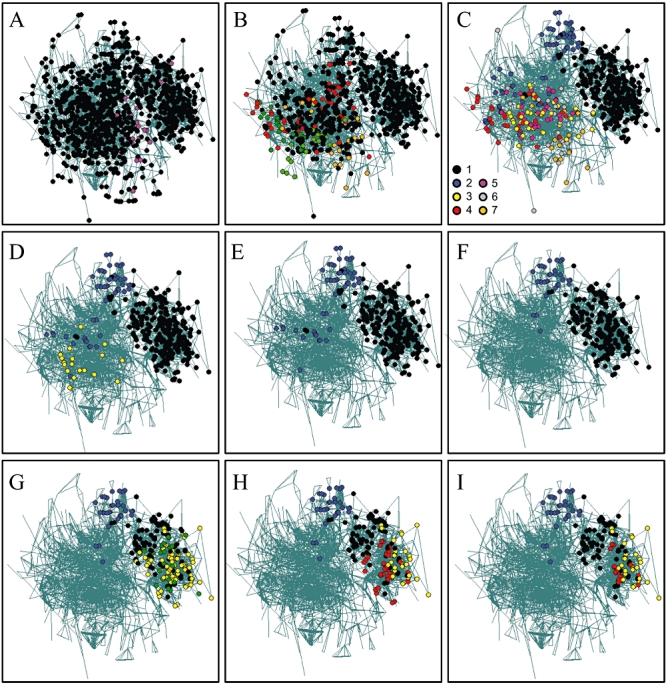
Identification of putative recombining groups by varying PSPB length (see Fig. 2A). As PSPB length was increased the giant component of the network broke down into smaller components (unbroken networks of sequences). Using the giant component of the network structure generated using 10 aa PSPBs as a framework, the positions of each of these smaller components was mapped. This was used as an approach to identifying putative recombining groups within the network. For clarity, only components containing 20 or more sequences are highlighted. PSPB lengths: (A) 12 aa (B) 13 aa (C) 14 aa (D) 15 aa (E) 16 aa (F) 17 aa (G) 18 aa (H) 19 aa (I) 20 aa. Components obtained using a PSPB length of 14 aa were numbered as shown in C and are referred to in the text as block-sharing groups 1–7.

As discussed above and shown in Fig. 2A, increasing the length of the PSPBs decreased the size of the largest unbroken network of sequences (the giant component). We used this observation to attempt to visualize putative groups of sequences that have recombined with one another or diverged relatively recently. Using the default network as a framework, we highlighted all the unbroken networks containing 20 or more sequences that were generated when different PSPB lengths between 12 and 20 aa were used (Fig. 4). At a PSPB length of 14 aa, only one of the *P. reichenowi* sequences remained attached to any *P. falciparum* sequences. Under these conditions (Fig. 4C) the small lobe of the giant component split from the large lobe. The resulting component, which we will henceforth refer to as block-sharing group 1 (Fig. 4C, black vertices), corresponded very well with the group A reference sequences (Fig. 3H). Ninety-five of 102 group A reference sequences lie in block-sharing group 1. In addition, 11/11 group A genes in the 3D7 genome fell in block-sharing group 1. As group A *var* are known to have a distinct chromosomal orientation that has been proposed to promote genetic structuring (Kraemer and Smith, 2003) the correspondence between block-sharing group 1 and group A supports the network as an approach to detecting distinct groups of recombining sequences.

The other main block-sharing group (block-sharing group 2, Fig. 4C, blue vertices) contained no known group A sequence despite containing many (58%) cys2 sequences of which 95% were from cys/PoLV group 2. The fact that cys/PoLV group 2 sequences are also common in block-sharing group 1 (19%) and make up 13% of the group A reference sequences suggests that cys/PoLV group 2 sequences may exist in distinct group A and non-group A forms.

This was of interest because we reported previously an association between expression of cys/PoLV group 2 sequences and the parasite rosetting phenotype (Bull *et al*., 2005). The rosetting phenotype, defined as the spontaneous binding of infected erythrocytes with uninfected erythrocytes, is associated with severe malaria and involves binding of DBLα domains to host complement receptor 1 (CR1; Rowe *et al*., 1997; Chen *et al*., 1998). In addition, we reported a sequence type called ‘sig2’ which was dominantly expressed in two parasite isolates from children with severe malaria (Bull *et al*., 2005). Sig2 sequences are in cys/PoLV group 2, and map to block-sharing group 2. We therefore asked whether the distinction between block-sharing groups 1 and 2 is maintained in an independent collection of sequences.

### Comparison with a worldwide data set of sequences

**Fig. 5:**
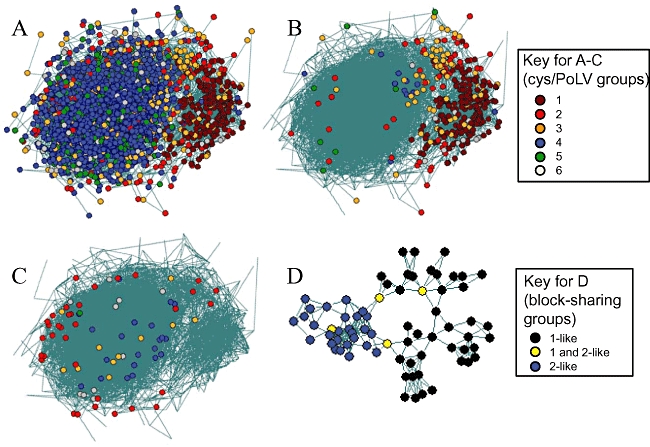
Comparison with sequences collected worldwide. A total of 2257 sequences collected worldwide were used to construct a new network. The vertices were coloured according to their cys/PoLV groups (A). A perl script (Folder S2) was used to identify sequences containing any of the 14 aa PSPBs carried by sequences within block-sharing groups 1 and 2. These were called ‘block-sharing group 1-like’ or ‘2-like’ sequences. Block-sharing group 1-like sequences are highlighted in (B). Block-sharing group 2-like sequences are highlighted in (C). The sequences matching these two sets of PSPBs tend to be located in different parts of the network. (This is much more clearly seen in 3D versions of the networks, see Folder S1.) (D) focuses on the cys/PoLV group 2 sequences. Vertices corresponding to hybrid sequences carrying PSPBs from both block-sharing groups 1 and 2 (‘1 and 2-like’) are coloured in yellow. Overall, fewer hybrid sequences occurred than would be expected by chance (see text).

To test whether the two block-sharing groups 1 and 2 defined above overlapped with sequences worldwide, we used an independent collection of 2257 sequences (See Table S2) from several sequencing projects (Fowler *et al*., 2002; Kirchgatter and del Portillo, 2002; Tami *et al*., 2003; Albrecht *et al*., 2006; Kyriacou *et al*., 2006; Barry *et al*., 2007; Kraemer *et al*., 2007; Montgomery *et al*., 2007; Normark *et al*., 2007), and constructed a network using the same set of conditions as before (Figs 2D and 3). Overall, the network (which we will call the ‘world network’) had similar features to the network from Kilifi (Fig. 5A).

To examine the correspondence with the block-sharing groups that we defined in Kilifi, we made a list of all the 14 aa PSPBs that were contained within sequences from block-sharing group 1 (137, 163, 133 and 140 different PSPBs 1, 2, 3 and 4 respectively) and block-sharing group 2 (26, 25, 30 and 32 different PSPBs 1, 2, 3 and 4 respectively). We then determined which sequences in the global network contained those 14 aa PSPBs. We called these sequences ‘block-sharing group 1-like’ and ‘block-sharing group 2-like’ (see Folder S2 for the perl script used for this search)

As shown in Fig. 5B–C, block-sharing group 1-like sequences were located almost exclusively within the small lobe of the network (Fig. 5B), whereas block-sharing group 2-like sequences fell exclusively within the large lobe (Fig. 5C). The distinction between these two groups of sequences is more clearly visible when the networks are viewed in three dimensions (see Folder S1).

As observed in the Kilifi network, cys2 sequences in cys/PoLV group 2 were common among both block-sharing group 1-like and 2-like sequences. Four of these cys/PoLV group 2 sequences are ‘hybrid’ sequences containing 14 aa PSPBs from both block-sharing groups 1 and 2 (Fig. 5D). This suggests that block-sharing group 1 and 2-like sequences can recombine with one another. To determine whether these two subgroups of cys/PoLV group 2 sequences are distinct, we determined whether ‘hybrid’ sequences occurred less than would be expected through random assortment. Of the 132 cys/PoLV group 2 sequences in the world network, 51 were block-sharing group 1-like, 31 were block-sharing group 2-like. The four ‘hybrid’ sequences occurred at a significantly lower frequency than expected by chance (Fisher's exact test *P* < 0.001), supporting the distinction between these sequences. See Table S3 and Fig. S7 for further analysis of block-sharing groups within the world network.

Included in the world network are 69 DBLα tags from fully sequenced *var* genes from HB3 and IT4. Figure S3 summarizes how 14 aa PSPBs from block-sharing groups identified in Fig. 4C correspond with the fully sequenced *var* genes from 3D7, HB3 and IT4. These data further support the distinction between block-sharing groups 1 and 2. However, none of the block-sharing group 2-like sequences from any of these isolates was in cys/PoLV group 2, suggesting that these sequences are not common to every parasite genome.

We explored the possibility that block-sharing group 2, cys/PoLV group 2 sequences might be geographically restricted. We first counted their frequency among sequences sampled from each continent. These were 0.61%, 0.85%, 1.27% and 2.2% in Papua New Guinea, South America, Asia and Africa respectively. Overall, significantly more of these sequences were sampled from Africa than non-African isolates (two-sided Fisher's exact test *P* = 0.015). The highest frequencies were from samples from Kenya (4.7%; Barry *et al*., 2007) and Uganda (4.2%; Normark *et al*., 2007). Though very preliminary, this observation suggests that block-sharing group 2, cys/PoLV group 2 sequences may be more prevalent in East Africa. No evidence for a bias toward Africa was observed for block-sharing group 1, cys/PoLV group 2 sequences (Fisher *P* = 0.48).

### Use of the network mapping approach to identify functional groups of genes

**Fig. 6:**
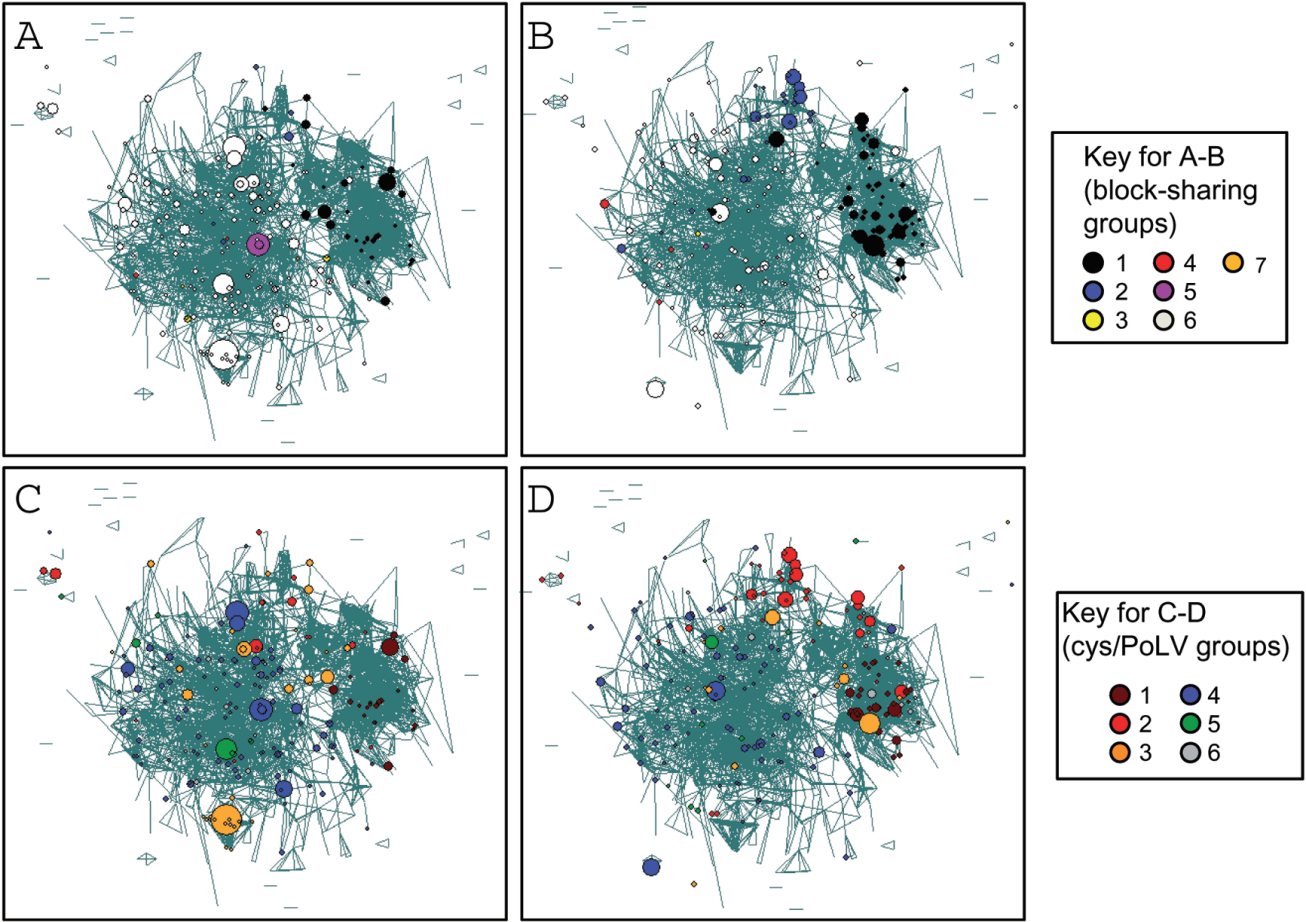
Comparisons of *var* gene expression in rosetting and non-rosetting isolates. Expression levels of each gene were assessed by sequencing multiple clones from a library of RT-PCR amplified DBLα sequences from parasite RNA (see Table S1). The percentage representation of each sequence was determined within each isolate. The mean percentage was then determined for two pools of isolates, one pool of seven parasite isolates with high rosetting (13–94%) and one pool of seven parasites with low rosetting (0–6%). The area of each vertex is proportional to this mean percentage representation. (A and C) Pooled data from seven parasite isolates exhibiting low rosetting. (B and D) Pooled data from seven parasite isolates exhibiting high rosetting. Vertices are coloured according to block-sharing groups obtained using PSPB length of 14 aa (A and B) and by cys/PoLV groups (C and D).

To further explore the distinction between cys/PoLV group 2 sequences falling in block-sharing groups 1 and 2, we re-analysed our existing rosetting frequency data by splitting the data according to block-sharing groups (Fig. 6).

*var* expression levels were estimated as described in *Experimental procedures*. Of the seven block-sharing groups defined in Fig. 4C, block-sharing groups 1 and 2 were the only ones for which there was evidence for an association between *var* expression and rosetting frequency (*r*_s_ = 0.629, *P* = 0.016 and *r*_s_ = 0.747, *P* = 0.0021 respectively). This was not surprising as no cys/PoLV group sequence fell in block-sharing groups 3–7. We further explored this association by breaking the block-sharing groups down by the cys/PoLV group of the sequence. There was evidence for an association between rosetting and cys/PoLV group 2 sequences from both block-sharing groups 1 and 2 (*r*_s_ = 0.73, *P* = 0.003; *r*_s_ = 0.69, *P* = 0.007 respectively). This raises the possibility that two distinct subgroups of cys/PoLV group 2 sequences are involved in parasite rosetting (see Fig. S4 for an alignment of these sequences).

While performing this analysis we found evidence for another group of rosetting sequences. These were those in both block-sharing group 1 and cys/PoLV group 6 (*r*_s_ = 0.86, *P* = 0.0001). Though this was unexpected, the sequences from this group that are expressed in rosetting parasite isolates are very similar and shares several unusual features with a sequence tag previously found to be associated with rosetting [the AFBR19 tag, GenBank ref. CAC41301 from the IT4 parasite line (Horrocks *et al*., 2004), see Fig. S4.

To visualize the differences between rosetting and non-rosetting isolates, Fig. 6 compares the aggregate expression levels of seven parasites with low rosetting frequencies with seven parasites with high rosetting frequencies. Mean expression scores within each set of seven isolates is proportional to the size of the vertices. The same data are shown for sequence classification by block-sharing group (Fig. 6A and B) and cys/PoLV group (Fig. 6C and D). [For an overall summary of the expression levels in all 21 isolates in relation to the cloning frequencies of each sequence from genomic DNA, see Fig. S5. For mapping of the groups of sequences found to be associated with rosetting and comparison with another recent study (Normark *et al*., 2007) see Fig. S6.]

Finally, as a preliminary test to see if cys/PoLV group 2 sequences might have a role in rosetting in parasites in other parts of Kenya we amplified DBLα tags from cDNA prepared from a rosetting parasite line isolated in Kisumu in western Kenya. The dominant sequence was identical to a block-sharing group 2, cys/PoLV group 2 sequence isolated in Kilifi (EMBL Accession No. CAJ40433.1, from rosetting isolate 4180, see Table S1 and Fig. S4). To explore the upstream region of this gene we developed two specific reverse primers (see *Experimental procedures*). Though we were unable to amplify sequence upstream using an upsA-specific forward primer, we successfully amplified products of the correct size using an upsB-specific forward primer. This further supports the idea that block-sharing group 2 sequences are non-group A sequences.

### Comparison with a phylogenetic approach

**Fig. 7:**
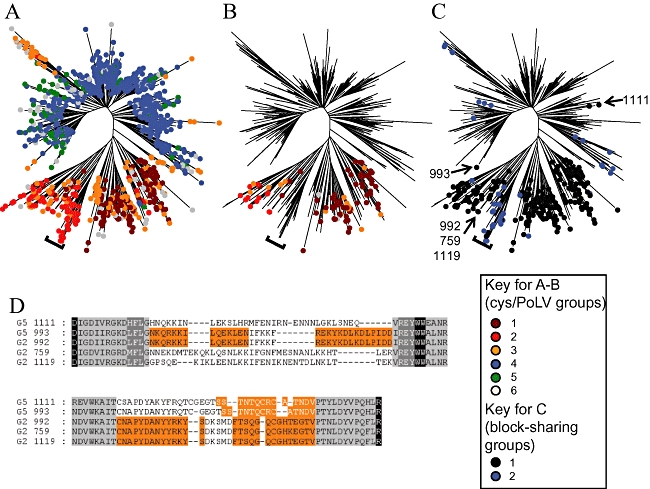
Comparison with a global sequence alignment. The sequences used to build the Kilifi network were aligned using the default parameters in MUSCLE. A. Sequences were coloured according the six cys/PoLV groups. B. Only group A reference sequences are shown (Trimnell *et al*., 2006). A region of the tree which lacks group A reference sequences is shown with a bracket. C. The positions of block-sharing group 1 (black) and 2 (blue) sequences are shown. There was a good correspondence between the block-sharing group 2 sequences and the gap in the group A reference sequences. Two block-sharing group 1 sequences (tags 993 and 1111) that fall outside the upsA region are indicated. D. Though tags 993 and 1111 are more like cys4 sequences they are linked to block-sharing group 1 because sequence 993 shares a PSPB with sequence 992.

We sought to determine how our approach compared with a phylogenetic approach that ignores the mosaic structure of the sequences. We chose the default parameters of the MUSCLE alignment algorithm because it separated well our previously defined cys/PoLV groups (Fig. 7A). As cys/PoLV groups were defined through an analysis of sequence length polymorphism without reference to phylogenetic trees (Bull *et al*., 2005), this provided a third independent approach to analysing the sequences. Figure 7B shows the position of the group A reference sequences. Figure 7C shows the positions of sequences falling into block-sharing groups 1 and 2. The majority of block-sharing group 2 sequences fall in a cluster that appears to fall among the group A reference sequences, but from Fig. 7B no group A reference sequences fall in this region of the tree. It would be hard to identify this cluster by observing Fig. 7A alone and it has no bootstrap support.

Figure 7C and D illustrates how the phylogenetic and network analyses can provide different information. Sequence tags 1111 and 993 are cys4 sequences. Both fall in block-sharing group 1 but appear distant from the other block-sharing group 1 sequences on the tree and from one another. This is because overall they align better with other cys4 sequences. Though sequences 1111 and 993 are dissimilar from one another they share a single block of sequence which links them together in the network. One of them (993) also shares a large block of sequence with a cys2 sequence tag 992.

## Discussion

Despite the apparently limitless diversity of *var* genes when sequences are aligned and globally compared (Barry *et al*., 2007), children growing up in endemic areas learn to recognize, after several years of exposure, the VSA expressed by parasites circulating in the parasite population (Barragan *et al*., 1998; Bull *et al*., 1998), and even variants from distant geographical regions (Aguiar *et al*., 1992; Nielsen *et al*., 2004). This suggests that there may be a limited number of important VSA types.

However, despite much research over the last 10 years it is still unclear how many antigenic variants of VSA exist or how to interpret *var* DNA sequence diversity data, in the absence of information on where the epitopes map. As a result, some reports have emphasized the potential importance of shared VSA epitopes and conserved *var* structure (Marsh and Howard, 1986; Aguiar *et al*., 1992; Bull *et al*., 2002; 2005; Nielsen *et al*., 2002; 2004; Kinyanjui *et al*., 2004), whereas others have tended to emphasize antigenic and sequence diversity (Forsyth *et al*., 1989; Newbold *et al*., 1992; Iqbal *et al*., 1993; Reeder *et al*., 1994; Barry *et al*., 2007).

The analysis presented here shows how extreme sequence diversity can be generated from blocks of sequence that are frequently shared between different molecules. The fact that even within a small region of *var*, sufficient connections exist to link all genes into a single network suggests that evolution of linear sequence by mutation may occur relatively slowly. This is supported by the localization of several *P. reichenowi* sequences within the network. Why would polymorphic regions be maintained over long time periods?

It is difficult to interpret this observation at present. Conservation of ancient polymorphic motifs has been observed previously in the MHC genes (Klein, 1987) and may suggest the presence of balancing selection on sequences that are under evolutionary constraints imposed by the need to maintain function while maintaining antigenic diversity (Ward *et al*., 1999). The presence of ancestral polymorphisms makes it difficult to know the age of the mosaic structure of the *var* sequences.

In our analysis we have attempted to make a distinction between older and more recent recombination events by altering the lengths of PSPBs. Using longer PSPBs the *P. reichenowi* sequences become separated from the *P. falciparum* sequences and smaller components of the network break off from the giant component. We have described these as ‘block sharing-groups’. The 14 aa PSPBs from cys/PoLV group 2, block-sharing group 2 sequences in the Kilifi network appear to be more prevalent in Africa than in other continents. Preliminary analysis of block sharing groups generated independently from the world network (Fig. S7) also show some suggestion of geographical structuring. However, it would be important to rule out the possibility that these apparent differences were generated by the slightly different PCR amplification conditions used in different studies.

By analysing the block-sharing groups present in the Kilifi network we hypothesized that a group of *var* genes associated with parasite rosetting (cys/PoLV group 2) may exist in both group A and non-group A forms. More detailed analysis of ups promoter regions upstream of large numbers of these genes is needed to confirm this. Similarity between the more conserved regions of group A and non-group A cys2 sequences might be maintained through shared function leaving the polymorphic regions to diversify independently. It will be of great interest in future studies to use the cys/PoLV group 2 genes to test the idea that genes with shared function exist in groups with distinct antigenic properties and independent expression control. The emergence of antigenically distinct variants of immune targets is predicted by previous studies. These studies suggest that antigenically distinct populations of immune targets that carry non-overlapping sets of multiple immune determinants can evolve in the face of immune selection pressure, even in the presence of recombination because hybrid molecules will be recognized by a greater number of hosts (McKenzie *et al*., 2001). The rationale for this was originally presented in a slightly different context (Gupta *et al*., 1996).

Ultimately, we aim to understand the relationships between DBLα tags to help us determine whether subsets of *var* genes are associated with severe malaria. A recent study in Uganda (Normark *et al*., 2007) has used a very different approach to ours. Instead of attempting to develop a system of classification these authors used an algorithm that searches directly for degenerate sequence motifs associated with rosetting and severe malaria from gapped alignments of DBLα sequences. It will be useful to determine whether similar sequences are identified using these different approaches (see Fig. S6 for a preliminary comparison of genes associated with parasite rosetting). A combination of different bioinformatic approaches together with large studies in various geographical settings are likely to be needed to uncover meaningful associations between specific *var* genes and syndromes of severe malaria. We hope this may lead to a better understanding of severe malaria and the identification of new targets of intervention.

## Experimental procedures

### Collection of clinical isolates

The majority of parasite samples used in the Kilifi network came from a study carried out at Kilifi District Hospital, situated in the east of Kenya, 50 km north of Mombasa on the coast. The hospital has a high-dependency ward to treat children with severe life-threatening malaria, a paediatric ward to treat children with moderate malaria and an outpatient department to treat children with mild malaria. Following informed consent, children were recruited if they had a primary diagnosis of malaria and parasitaemia of one trophozoite per 100 uninfected erythrocytes. Isolates were collected and white blood cells removed as described previously (Bull *et al*., 2000). Parasites were collected from children attending hospital between July 1998 and February 1999.

### Sequences used in this study

*var* sequences from Kilifi were sequenced from cDNA and genomic DNA libraries generated as described previously (Bull *et al*., 2005). Some of these (878 non-identical sequences from isolates 4162, 4172, 4168, 4013, 4130, 4178, 4142, 4180, 4187, 4140, 4161, 4129) have been presented previously (Bull *et al*., 2005). Sequences from a further nine severe isolates (4014, 4015, 4018, 4021, 4028, 4030, 4037, 4038, 4059) were also included in the analysis (350 non-identical sequences, see below and Table S1). *P. reichenowi* sequences (29) were downloaded from the Sanger website (http://www.sanger.ac.uk). Group A reference sequences (102) are from Trimnell *et al*. (2006). 3D7 sequences (61) are from Gardner *et al*. (2002). Two main networks were analysed in this study. The ‘Kilifi network’ contains 1420 sequences: 1228 sequences from Kilifi, 29 *P. reichenowi*, 102 group A reference, 61 3D7. There was some overlap in the sequences from these different sources. As this number was a small we chose for this analysis to leave these sequences as distinct vertices. The world network contains 2257 sequences from different locations worldwide, excluding Kilifi (see Table S2).

### Construction of networks

An Excel spreadsheet (Microsoft) was developed which extracts four blocks of amino acids from specific windows of DBLα sequence tags defined using three anchor points. Default positions set for the PSPBs were as follows (see vertical arrows in Fig. 1B): the 5′ amino acid of PSPB1 was set 15 aa from the 5′ of the tag region; the 3′ end of PSPB2 was fixed 5 aa 5′ to the conserved central WW motif, the 5′ end of PSPB3 was fixed at 13 aa 3′ to the central WW motif; the 3′ end of PSPB4 was fixed 13 aa from the 5′ end of the tag region. Standard Excel spreadsheet functions were used to determine which sequences shared PSPBs and to format this information for import into a network analysis package (Pajek, see below).

### Visualization of networks

Networks were drawn and visualized using freely available software: Pajek was used for initial construction and analysis of the networks (V. Batagelj, A. Mrvar: Pajek – Program for Large Network Analysis. http://vlado.fmf.uni-lj.si/pub/networks/pajek/). 2D networks were drawn using the Kamada Kawai algorithm (Kamada and Kawai, 1989). 3D networks were drawn using the Fruchterman Reingold algorithm (Fruchterman and Reingold, 1991) within Pajek. 3D networks were exported as *.wrl files (see Folder S1) and can be visualized using Cortona virtual reality modelling language client 4.2 software (http://www.parallelgraphics.com or http://software.filefactory.com). Each *var* sequence represented a vertex within the network. An edge was formed between two vertices if they shared one or more PSPBs region. In the present analysis no weighting was given to edges with respect to the number of PSPBs shared. Visualization of the divisions of the sequences into cys/PoLV groups and block-sharing groups was achieved through formatting the data as Pajek partition files. Visualization of *var* gene expression data (see below) was achieved by formatting the data as Pajek vector files. See Folder S3 for the Kilifi network in Pajek format.

### Cys/PoLV sequence grouping

Sequences were initially classified using positions of limited variability (PoLV, Fig. 1B) as described previously (Bull *et al*., 2005; 2007). Three features were used to group the sequences into one of six ‘cys/PoLV groups’. These are (i) the position of limited variability1 (PoLV1) motif situated at the 3′ end of homology block D; (ii) the PoLV2 motif situated at the 5′ end of homology block F; and (iii) a count of the number of cysteine residues within the tag sequence. Groups were defined as follows: *group 1*: MFK* motif at PoLV1, 2 cysteines; *group 2*: *REY motif at PoLV2, 2 cysteines; *group 3*: 2 cysteines, not group 1 or 2; *group 4*: 4 cysteines, not group 5; *group 5*: *REY motif at PoLV2, 4 cysteines; *group 6*: presence of 1, 3, 5 or 6 cysteines. The asterisk ‘*’ denotes any amino acid. MFK* motifs at PoLV1 and *REY motifs at PoLV2 are mutually exclusive in tag sequences isolated worldwide (Bull *et al*., 2007).

### Analysis of expression patterns in relation to parasite rosetting

Our approach to assessing *var* gene expression has been described previously (Bull *et al*., 2005). After preparing cDNA libraries of DBLα tags prepared from each isolate, following transformation into *E. coli*, either 48 or 96 colonies were picked at a time at random and sequenced. For each parasite isolate, and following sequence quality control (previously described: Bull *et al*., 2005), the number of successful sequences that fell into each category (e.g. cys/PoLV group or block-sharing group) was counted and expressed as a percentage of the total number of sequences obtained for that isolate. These expression scores were compared between isolates in relation to the rosetting frequency scores obtained from a total of 14 isolates. These included 12 isolates previously described (Bull *et al*., 2005) together with two additional isolates from Kilifi (see Table S1). The correlation between *var* expression within each defined group and rosetting frequency was determined using Spearman's rank correlation coefficient (*r*_s_). Rosetting assays were performed as described previously (Bull *et al*., 2000). Rosetting frequency was scored by counting the percentage of infected erythrocytes that are bound to two or more uninfected erythrocytes.

### Searching for PSPBs within the sequences collected worldwide

The 14 aa PSPBs from block-sharing group 1 and 2 genes were used to search Fasta files of sequences for hits to any of the PSPBs associated with that block-sharing group (see Folder S2 for the Perl script used). To test for overlap in genes containing 14 aa PSPBs from block-sharing groups 1 and 2, we counted the number of cys/PoLV group 2 genes from the world *var* network that matched PSPBs from block-sharing group 1 only, the number that matched PSPBs from block-sharing group 2 only, the number that matched PSPBs from both block-sharing group 1 and 2 and the number that did not match any. These numbers were expressed as a 2 × 2 table, and Fisher's two-sided exact test was used to determine whether there were less sequences that matched both block-sharing group 1 and 2 PSPBs than would be expected by chance.

### Global sequence alignment and tree construction

Sequences were aligned using MUSCLE (Edgar, 2004) using default parameters. Neighbour-joining trees were constructed using MEGA3.1 (Kumar *et al*., 2004). Alignments in Fig. S4 were visualized using Genedoc (http://www.nrbsc.org/gfx/genedoc/index.html).

### Characterization of a block-sharing group 2 gene from a rosetting isolate from Kisumu

DBLα tag sequences were amplified from cDNA synthesized from RNA extracted from the SA075 parasite line, as described previously (Bull *et al*., 2005). RNA extraction was performed on purified ring-infected erythrocytes from synchronized cultures following long-term maintenance of the rosetting phenotype by using a percoll gradient. Following transformation, a total of 37 colonies were picked tags sequenced. Seventeen of the tag sequences had a sequence identical to a sequence previously isolated in Kilifi. Reverse primers were developed both to a LYLD motif at the 5′ end [PoLV1(LYLD), TTCATGATCAAGGTATAAATC] and a PTNL motif at the 3′ end [PoLV4(PTNL), ACGTAATCTAAATTGGTAG]. Each was tested for amplification of cDNA using previously described upsA- and upsB-specific forward primers (upsA750: AACATKGTTCTATTTTCTC and upsB: TTGCCTCTDTTGTTATCTC) (Bull *et al*., 2005). Following 35 cycles with denaturation temperature 94°C, annealing temperature 47°C and extension temperature 65°C in the presence of Platinum *Taq* DNA polymerase High Fidelity (Invitrogen), the products obtained using the upsB primer were sequenced to confirm the identity of the DBLα tag region. These products were 1.4 kb and 1.6 kb for the PoLV1(LYLD) and PoLV4(PTNL) primers respectively.

## Supplementary material

This material is available as part of the online article from: http://www.blackwell-synergy.com/doi/abs/10.1111/j.1365-2958.2008.06248.x

(This link will take you to the article abstract).
