# Supplementary material for: Plasmodium falciparum antigenic variation. Mapping mosaic var gene sequences onto a network of shared, highly polymorphic sequence blocks
Source: Mol Microbiol. 2008 Jun;68(6):1519–34. doi: 10.1111/j.1365-2958.2008.06248.x (PMC2440560; doi:10.1111/j.1365-2958.2008.06248.x)
Supplement: Supplementary file 1 [file mmi0068-1519-SD1.pdf]

Supporting Information

Figure S1. ***Networks drawn with only three PSPBs***. These were drawn to demonstrate that clustering within the Kilifi network is not controlled only by single PSPBs. The networks were drawn using the same 1420 sequences. A-C) PSPB4 removed. D-F) PSPB1 removed. G-H) PSPB3 removed. J-L) PSPB2 removed. In each case the size of the giant component of the network was reduced but the clustering of groups within the network was maintained. The clustering of previously established groups is not simply due to the close association of PoLV motifs with PSPB motifs. Cys/PoLV group 1 is defined in part by an MFK* motif at PoLV1 next to PSPB1. Clustering of group 1 is maintained when PSPB1 is removed (D). Cys/PoLV group 2 is defined in part by an *REY motif at PoLV2 next to PSPB2. Clustering of group 2 is maintained when PSPB2 is removed (J). A,D,G,J) Vertices are coloured according to cys/PoLV groups. B,E,H,K) only group A reference sequences are indicated. C,F,I,L) vertices are coloured according to whether they fell in block-sharing group 1 or 2.

Figure S2. ***Sharing of PSPBs between cys/PoLV groups***. The length of coloured bars is proportional to the percentage of different PSPB variants that are shared between cys/PoLV groups 1-5. The length of the bars is set according to the scale shown on the left. Each percentage was calculated with respect to the total number of PSPB variants existing at each position. Colours correspond to the PSPBs 1-4. Numbers in circles indicate the cys/PoLV groups. Dotted lines highlight each between group comparison. Note the absence of any PSPBs shared between groups 1 and 5 and the deficiency of PSPB2 shared between groups 4 and 5. Group 6 was excluded because it appears to result from recombination of a very broad range of molecules. Note the sharing of PSPBs between cys2 and cys4 sequences (especially between cys/PoLV groups 3 and 4).

Figure S3. ***Matching of var sequences from the 3D7, HB3 and IT4 genomes with 14aa PSPBs from block-sharing groups 1-7 defined within the 14aa PSPB network*** (Figure 4C). Gene classifications are from {Kraemer, 2007 #1289}. Block-sharing groups 5 and 6 are combined because one of the sequences from HB3 carried PSPBs from both these block-sharing groups. Note the tendency of this combined block-sharing group to carry PSPBs that match central *var* genes (group BC or C).

Figure S4. ***Multiple alignment of sequences associated with parasite rosetting.***
Sequences were selected for alignment if they were expressed in the seven rosetting parasite isolates (see Figure 6 B and D) and fell in sub-groups that showed evidence for an association with parasite rosetting. The sub-groups were cys/PoLV group 2 sequences in block-sharing group 1 (“1/2”), cys/PoLV group 6 sequences in block-sharing group 1 (“1/6”) and cys/PoLV group 2 sequences in block-sharing group 2 (“2/2”).

Figure S5. ***Comparisons of var gene expression in different clinical parasite isolates***. Expression levels of each gene were assessed by sequencing multiple clones from a library of RT-PCR amplified DBLα sequences from RNA (see Table S1). The percentage representation of each sequence was determined within each isolate. The mean percentage was then determined for all 21 parasite isolates. The area of each node is proportional to this mean percentage representation. Colony picking from clones generated from cDNA (A and C) were compared with sequences obtained by amplifying genomic DNA (B and D). Each graph is coloured either using block-sharing groups (A and B) or cys/PoLV groups (C and D).

Figure S6. ***Comparison with the approach of Normark et al. 2007.*** A recent study in Uganda {Normark, 2007 #1422} suggests that it may be possible to search for functional motifs directly using a specifically designed motif detection algorithm. Three sets of related motifs, H1, H2 and H3 were identified that showed an association with rosetting. A) The figure indicates the positions of sequences within the Kilifi network that contained the identified motifs at any position. The degenerate motifs comprise of the following specific motifs used in our search H1: RYSANI, FSKNI; H2: TCAAKV, TCDATM, TCGATM, TCGATV, TCKAEV ; H3: DDKVQK, DKVEKG, EDKVQK, HDAVEK, KDAVQK, KDAVQN, KDDVEK, KDEVKE, NDEVWK. The mapping of the sequences on the network suggest that these motifs are associated with non-overlapping groups of sequences that have some correspondence to those identified in Kilifi. B) indicates the positions of sequences that, as groups, exhibited a significant correlation between expression levels and parasite rosetting frequency in our study (see text) "1/2"= block-sharing group 1, cys/PoLV group 2; "2/2"= block-sharing group 2, cys/PoLV group 2 ; "1/6"= block-sharing group 1, cys/PoLV group 6. C and D) compares the expression levels of sequences parasites exhibiting low (C) and high (D) levels of rosetting, as described in Figure 6 but coloured according to the Nomark groups.

Figure S7. ***Exploration of geographical structuring of var sequences.*** A) Barry *et al.* {Barry, 2007 #1327} have recently reported evidence for geographical structuring of *var* sequences in Papua New Guinea (PNG) using a collection of sequences sampled from 30 parasites in 1999. These sequences are broadly distributed over the world network. This may be because the intra-genomic diversity of var sequences sampled within each genome is maintained throughout the world. One approach to exploring geographical structuring using the network approach is to explore the composition of block-sharing groups. B) shows an example. This figure shows the 7 block-sharing groups containing 20 or more sequences that were generated using a PSPB length of 20 amino acids. C) Considering only the sequences sampled in these 7 groups, the percentage of sequences falling in each block-sharing group is expressed as a percentage of the total number of sequences sampled from each continent. There appears to be an excess of Asian sequences in block-sharing groups 2 and 4 and a deficiency in block-sharing group 1, and an excess of sequences from PNG in block-sharing group 3 (all Fishers exact, 2-sided $P<0.001$). Note that the location of the 7 main block sharing groups is different to the main groups found in the Kilifi network using 20 aa PSPB (Figure 4I) which tended to be located within or next to the small lobe. The difference may be attributed to the geographic heterogeneity of the sequences in the world network, their bias towards sequences amplified from genomic DNA and the absence of group A reference sequences.

Folder S1.zip. Zipped file containing three dimensional networks. See materials and methods for more details.

Folder S2.zip. Perl script for classifying sequences by cys/PoLV group and identifying block sharing group 1 and 2-like sequences (i.e. those that share 14 amino acid PSPBs with block sharing groups 1 and 2). This script is a modified version of one we described previously and also classifies DBLα tag sequences into cys/PoLV groups (see Bull *et al.* 2007, Mol. Biochem. Parasitol (54) 98-102 for details)

Folder S3.zip. Pajek project file for the Kilifi network. This contains data on the sequences in Dataset S1

Table S1. Clinical parasite isolates used in the study.

Table S2. Sequences used in the world network.

Table S3. Sharing of 14aa PSPB in the world network.

Dataset S1. The 1420 sequences and sets of PSPBs used in the Kilifi network.

Figure S1

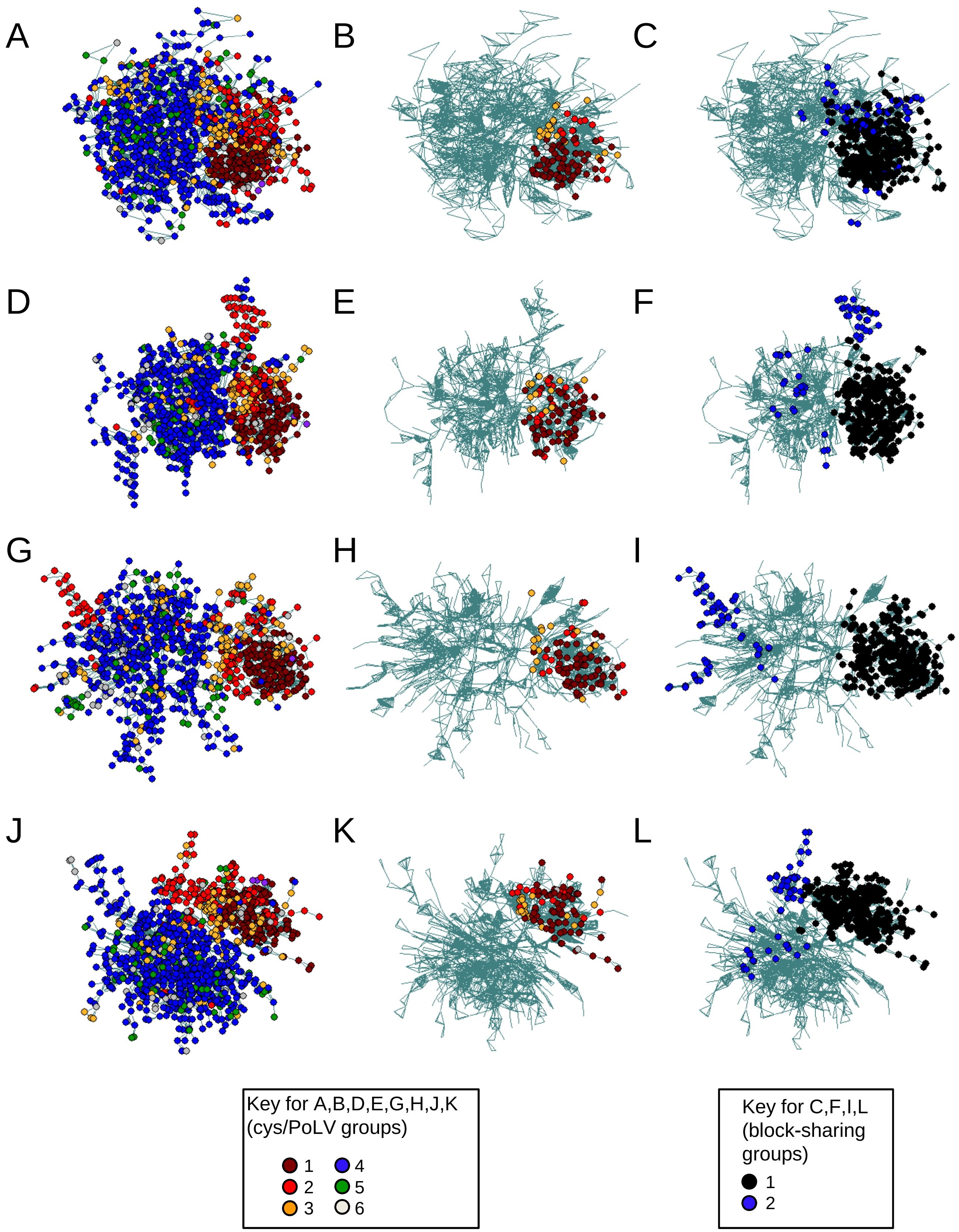

A
B
C
D
E
F
G
H
I
J
K
L
Key for A,B,D,E,G,H,J,K (cys/PoLV groups)
1
2
3
4
5
6
Key for C,F,I,L (block-sharing groups)
1
2

Figure S2

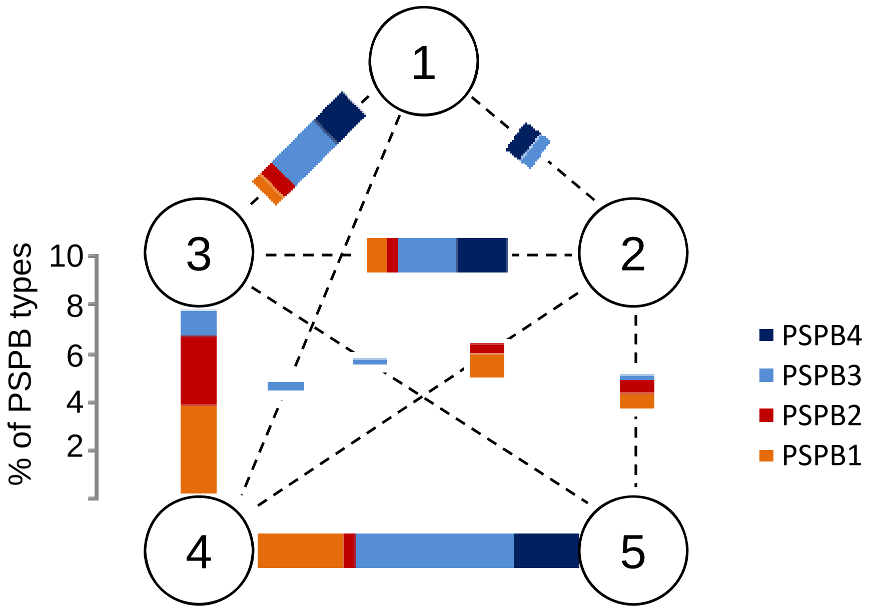

Figure S3

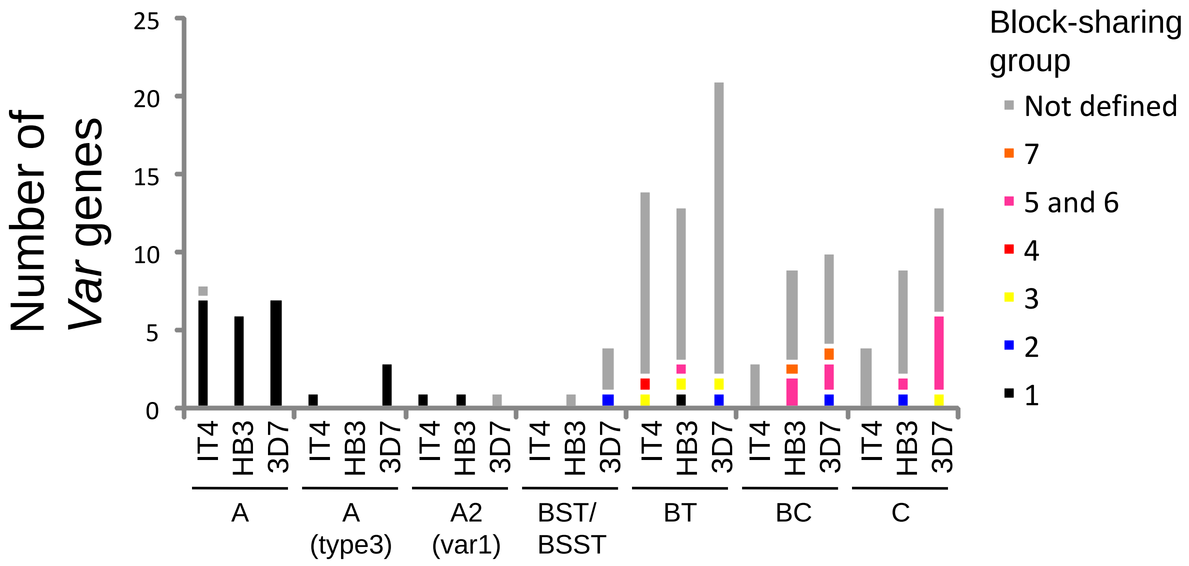
Number of
Var genes
25
20
15
10
5
0
IT4
HB3
3D7
A
A
(type3)
A2
(var1)
BST/
BSST
BT
BC
C
Block-sharing group
Not defined
7
5 and 6
4
3
2
1

Figure S4

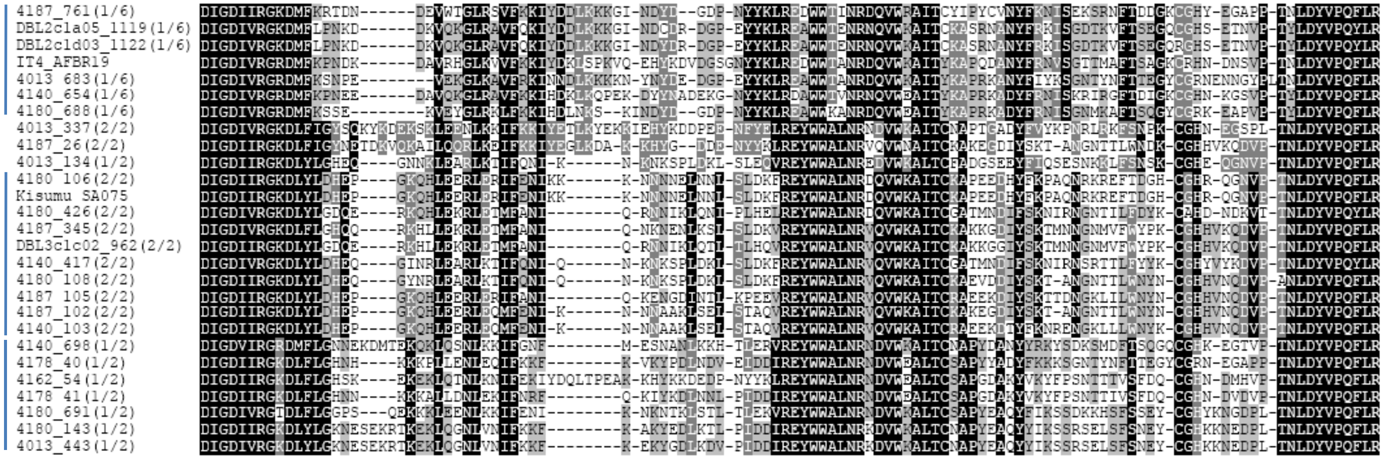
4187_761(1/6)
DBL2c1a05_1119(1/6)
DBL2c1d03_1122(1/6)
IT4_AFBR19
4013 683(1/6)
4140_654(1/6)
4180_688(1/6)
4013_337(2/2)
4187_26(2/2)
4013_134(1/2)
4180 106(2/2)
Kisumu SA075
4180_426(2/2)
4187_345(2/2)
DBL3c1c02_962(2/2)
4140_417(2/2)
4180 108(2/2)
4187 105(2/2)
4187_102(2/2)
4140_103(2/2)
4140_698(1/2)
4178_40(1/2)
4162_54(1/2)
4178 41(1/2)
4180_691(1/2)
4180_143(1/2)
4013_443(1/2)

Figure S5

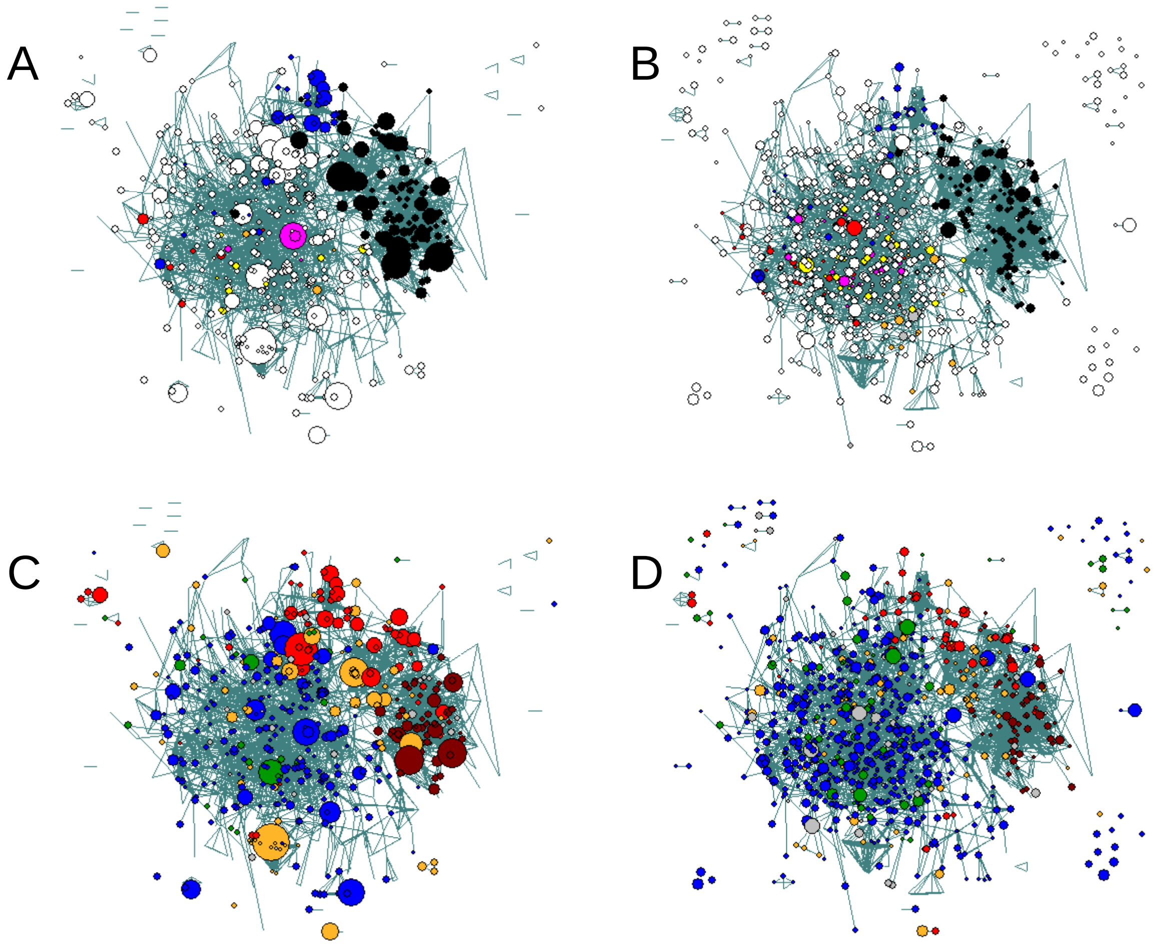

Figure S6

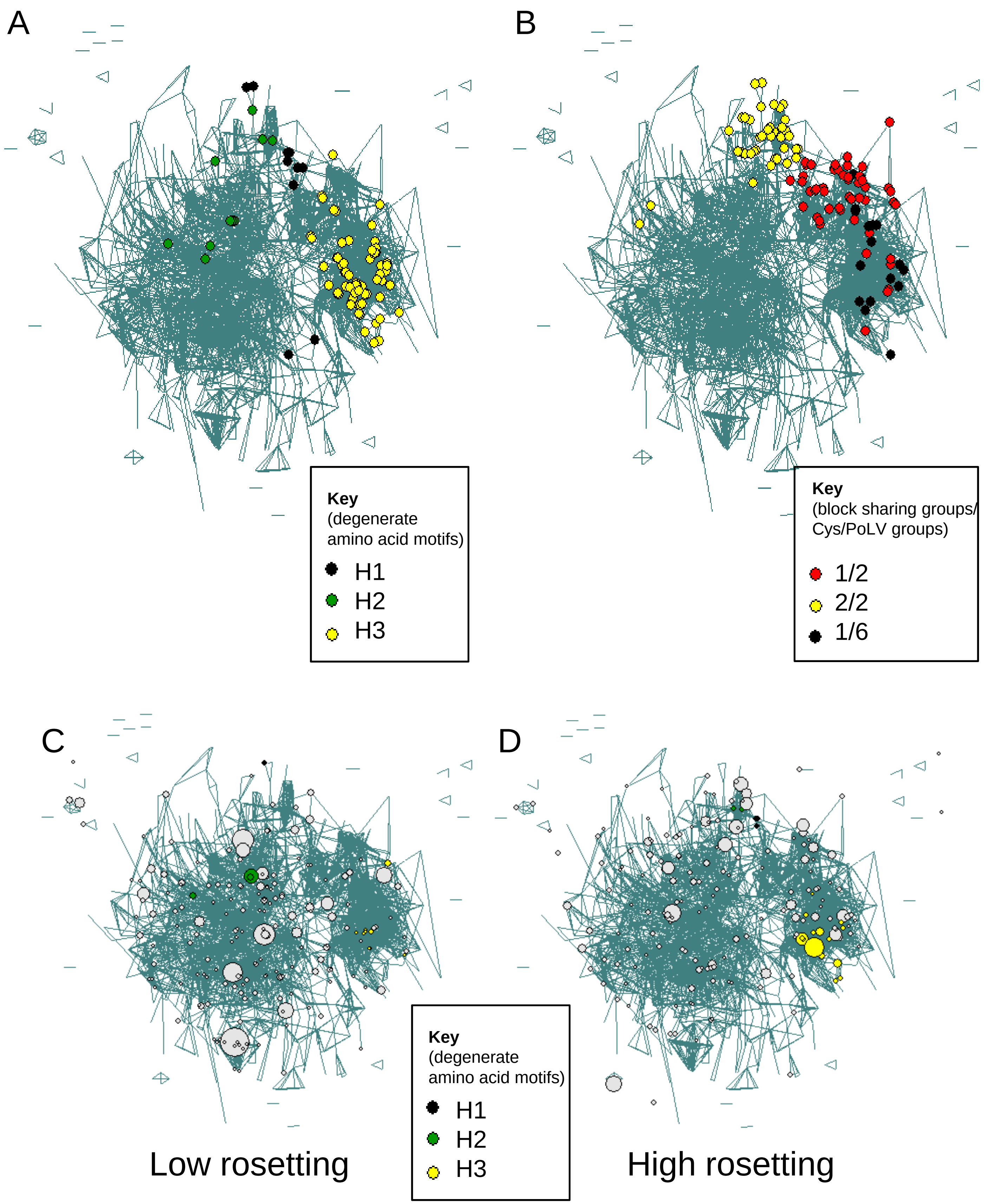
A
B
Key
(degenerate
amino acid motifs)
H1
H2
H3
Key
(block sharing groups/
Cys/PoLV groups)
1/2
2/2
1/6
C
D
Low rosetting
Key
(degenerate
amino acid motifs)
H1
H2
H3
High rosetting

Figure S7

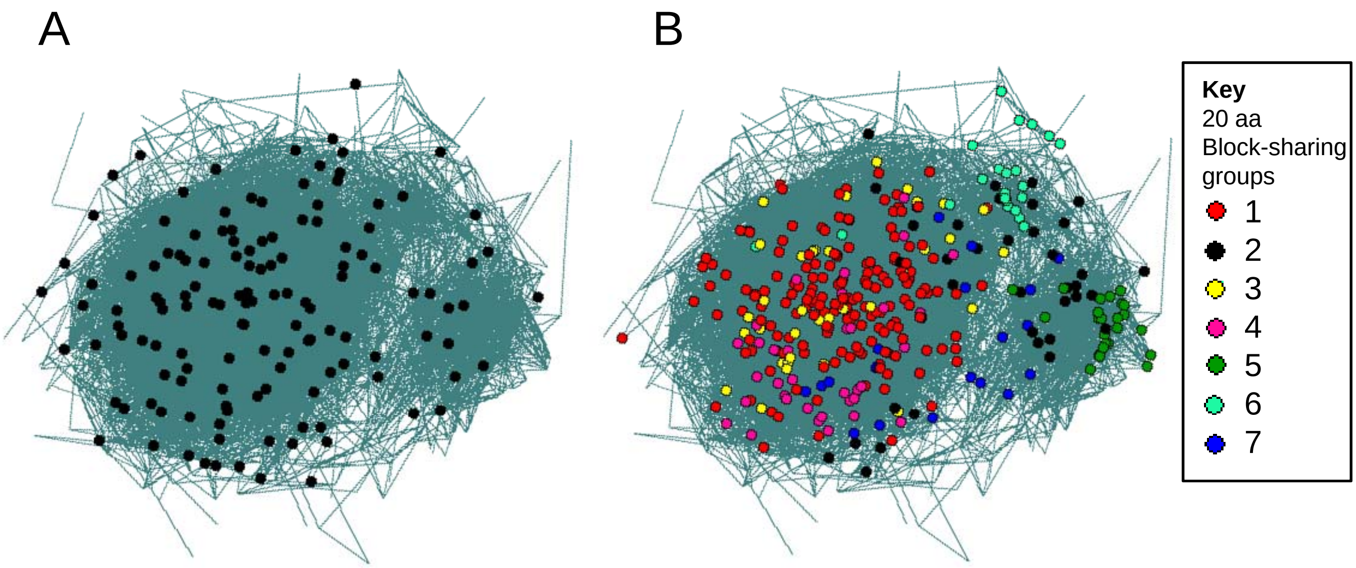

A
B
Key
20 aa
Block-sharing
groups
1
2
3
4
5
6
7

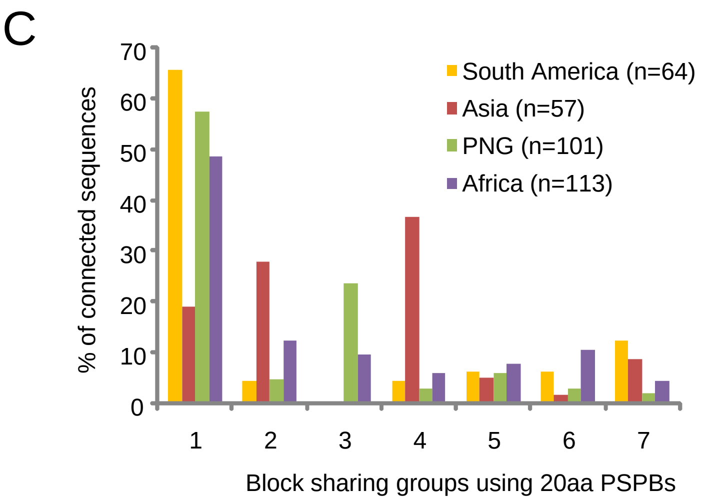

C
70
60
50
40
30
20
10
0
% of connected sequences
South America (n=64)
Asia (n=57)
PNG (n=101)
Africa (n=113)
1
2
3
4
5
6
7
Block sharing groups using 20aa PSPBs

Table S1. ***Clinical parasite isolates used in the study***

Isolate	cDNA clones[a]	Disease status[b]	% rosetting[c]	Age (months)
4013	81	Severe	High (22)	24
4014	34	CM	-	20
4015	41	Sev Anaemia/CM	High (40)	28
4018	40	Severe	High (13)	46
4021	42	CM	-	32
4028	39	CM	-	33
4030	7	CM	-	53
4037	34	CM	-	54
4038	39	Sev Anaemia	-	5
4059	25	Severe	-	25
4129	30	Mild	Low(5)	-
4130	110*	Mild	Low(6)	8
4140	33	Severe	High (94)	19
4142	41	Sev Anaemia	Low (4)	40
4161	123*	Severe	Low (0)	64
4162	26	CM	Low (0)	55
4168	73	Mild	Low (2)	85
4172	42	Mild	Low (1)	25
4178	69*	Mild	High (20)	41
4180	31	Mild	High (46)	4
4187	63*	Severe	High (56)	84

[a] number of cDNA clones successfully sequenced to assess expression (* the sequences were pooled from two ligations and transformations)
[b]CM=Cerebral malaria (Blantyre score <3); Sev Anaemia=severe anaemia (<5g/dL haemoglobin); Mild: not admitted to hospital; Severe= severe malaria without severe anaemia or cerebral complications.
[c] percentage of parasite infected erythrocytes forming rosettes (adherence to at least 2 uninfected erythrocytes)

Table S2. Sequences used in the world network

Region	Number of sequences	Reference
Brazil	37	(Barry et al., 2007)
Brazil	41	(Kirchgatter and del Portillo H, 2002)
Gabon	658	(Barry et al., 2007) and Barry (Unpublished)
India	18	(Barry et al., 2007)
Indochina	27	(Barry et al., 2007)
Kenya	43	(Barry et al., 2007)
Lab isolate HB3	36	(Kraemer et al., 2007)
Lab isolate IT4	33	(Kraemer et al., 2007)
Malawi	74	(Montgomery et al., 2007)
Mali	124	(Kyriacou et al., 2006)
Phillipines	53	(Fowler et al., 2002)
PNG	326	(Barry et al., 2007)
PNG	22	(Fowler et al., 2002)
PNG	140	(Kaestli et al., 2004)
Sierra Leone	43	(Barry et al., 2007)
Solomon Is	70	(Fowler et al., 2002)
South America	107	(Albrecht et al., 2006)
Thailand	69	(Barry et al., 2007)
Uganda	241	(Normark et al., 2007)
Venezuela	49	(Tami et al., 2003)
Zimbabwe	46	(Barry et al., 2007)

Albrecht, L., Merino, E.F., Hoffmann, E.H., Ferreira, M.U., de Mattos Ferreira, R.G., Osakabe, A.L., Dalla Martha, R.C., Ramharter, M., Durham, A.M., Ferreira, J.E., Del Portillo, H.A., and Wunderlich, G. (2006) Extense variant gene family repertoire overlap in Western Amazon Plasmodium falciparum isolates. Mol Biochem Parasitol 150: 157-165.

Barry, A.E., Leliwa-Sytek, A., Tavul, L., Imrie, H., Migot-Nabias, F., Brown, S.M., McVean, G.A., and Day, K.P. (2007) Population Genomics of the Immune Evasion (var) Genes of Plasmodium falciparum. PLoS Pathog 3: e34.

Fowler, E.V., Peters, J.M., Gatton, M.L., Chen, N., and Cheng, Q. (2002) Genetic diversity of the DBLalpha region in Plasmodium falciparum var genes among Asia-Pacific isolates. Mol Biochem Parasitol 120: 117-126.

Kaestli, M., Cortes, A., Lagog, M., Ott, M., and Beck, H.P. (2004) Longitudinal assessment of Plasmodium falciparum var gene transcription in naturally infected asymptomatic children in Papua New Guinea. J Infect Dis 189: 1942-1951.

Kirchgatter, K., and del Portillo H, A. (2002) Association of severe noncerebral Plasmodium falciparum malaria in Brazil with expressed PfEMP1 DBL1a sequences lacking cysteine residues. Mol Medicine 8: 16-23.

Kraemer, S.M., Kyes, S.A., Aggarwal, G., Springer, A.L., Nelson, S.O., Christodoulou, Z., Smith, L.M., Wang, W., Levin, E., Newbold, C.I., Myler, P.J., and Smith, J.D. (2007) Patterns of gene recombination shape var gene repertoires in Plasmodium falciparum: comparisons of geographically diverse isolates. BMC Genomics 8: 45.

Kyriacou, H.M., Stone, G.N., Challis, R.J., Raza, A., Lyke, K.E., Thera, M.A., Kone, A.K., Doumbo, O.K., Plowe, C.V., and Rowe, J.A. (2006) Differential var gene transcription in Plasmodium falciparum isolates from patients with cerebral malaria compared to hyperparasitaemia. Mol Biochem Parasitol 150: 211-218.

Montgomery, J., Mphande, F.A., Berriman, M., Pain, A., Rogerson, S.J., Taylor, T.E., Molyneux, M.E., and Craig, A. (2007) Differential var gene expression in the organs of patients dying of falciparum malaria. Mol Microbiol 65: 959-967.

Normark, J., Nilsson, D., Ribacke, U., Winter, G., Moll, K., Wheelock, C.E., Bayarugaba, J., Kironde, F., Egwang, T.G., Chen, Q., Andersson, B., and Wahlgren, M. (2007) PfEMP1-DBL1alpha amino acid motifs in severe disease states of Plasmodium falciparum malaria. Proc Natl Acad Sci U S A 104: 15835-15840.

Tami, A., Ord, R., Targett, G.A., and Sutherland, C.J. (2003) Sympatric Plasmodium falciparum isolates from Venezuela have structured var gene repertoires. Malar J 2: 7.

Table S3. Sharing of 14aa PSPB in the world network.

	BS1	BS2	BS3	BS4	BS5	BS6	BS7
BS1	322	5*	0***	0*	0**	0**	1**
BS2		81	1	1	0	0	2
BS3			71	0	1	1	2
BS4				25	0	1	0
BS5					51	1	1
BS6						49	3
BS7							67

Numbers on the diagonal show the number of sequences in the world network that contain 14aa PSPB from block-sharing groups 1-7 (BS1-BS7, see Figure 4C). Shown in the off-diagonal positions are the numbers of sequences that contain 14aa PSPB from two different block sharing groups. We tested whether these “hybrid” sequences occurred less often than expected by chance. The asterisks show the significance of this test. Fishers 2-sided exact test: P<0.05*; P<0.01**; P<0.001***. Overall, this analysis shows that the block-sharing groups tend to coalesce as more sequences are added to the network. However, there remains a clear distinction between sequences containing PSPB from block sharing group 1 and those that contain PSPB from other block-sharing groups. As shown in Figure S7 this picture may be complicated by geographical structuring. It would be interesting in further studies to explore the coalescence of block-sharing groups in a larger network of sequences from Kilifi.
